# Beidseitige retinale Blutungen nach „acute respiratory distress syndrome“ (ARDS) bei COVID-19-Pneumonie

**DOI:** 10.1007/s00347-022-01676-6

**Published:** 2022-07-04

**Authors:** P. Peschke, F. Weinand

**Affiliations:** Klinik für Augenheilkunde, BundeswehrZentralkrankenhaus Koblenz, Rübenacher Str. 170, 56072 Koblenz, Deutschland

## Anamnese

Ein 33-jähriger, männlicher Patient wurde aufgrund starker Sehminderung beidseits bei Zustand nach ARDS im Rahmen einer COVID-19-Pneumonie unserer Klinik zugewiesen. Im Vorfeld wurde bei progredientem Verlauf einer COVID-19-Erkrankung die stationäre Aufnahme, Intubation und kontrollierte Beatmung notwendig. Im Verlauf stellte sich ein ARDS bei COVID-19-Pneumonie (ARDS Stufe 3 nach Berlin-Klassifikation) ein. Insgesamt wurde der Patient unter intermittierender Bauchlagerung und Picco-gesteuerter Katecholamin- und Volumenersatztherapie 48 Tage lang beatmet. Nach erstmaligem Wiedererlangen der Artikulationsfähigkeit gab der Patient eine beidseitige Visusminderung an (rechts > links). Ein augenärztlicher Kollege stellte beidseitig Netzhaut- sowie Glaskörperblutungen fest und empfahl eine zeitnahe Evaluierung bzw. operative Sanierung in einer Augenklinik. Eine Brille hat der Patient bei zuvor gutem Visus nie getragen. Augenerkrankungen, -operationen oder -traumata im Vorfeld wurden verneint, die Familienanamnese war leer. Nebst Adipositas per magna bestanden ansonsten keine Vorerkrankungen und keine Dauermedikation.

## Befund

**Figure 1:**
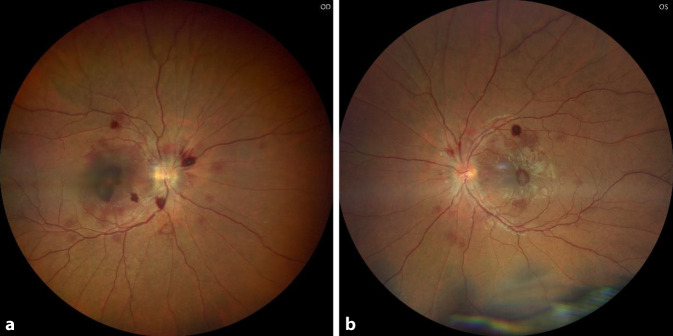

Bei Übernahme zeigten sich am Gesicht multifokale, dermale Druckläsionen im Rückbildungsstadium, die Lider waren reizfrei. Es wurde ein initialer Visus von rechts Handbewegungen (sine correctione, Gläser bessern nicht) und links 0,6 (sc, Glbn, mit multifokalen Gesichtsfeldskotomen) festgestellt. Neben unauffälligen vorderen Augenabschnitten zeigten sich fundoskopisch und OCT-morphologisch (optische Kohärenztomographie) rechts auf dem Boden einer hämorrhagischen Glaskörpertrübung eine foveale Blutung mit subretinalem Anteil sowie mehrere peripapilläre Blutungen. Links fanden sich im Bereich der Gefäßbögen disseminierte Sub-ILM-Blutungen und eine foveale Blutung (Abb. 1 und 2).

**Figure 2:**
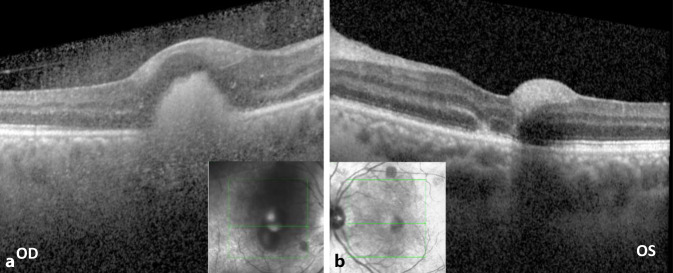

## Diagnose

Beidseitige Valsalva-Retinopathie mit subretinalen und Sub-ILM-Anteilen nach Überdruckbeatmung in Bauchlage (ARDS bei COVID-19-Pneumonie).

## Therapie und Verlauf

**Figure 3:**
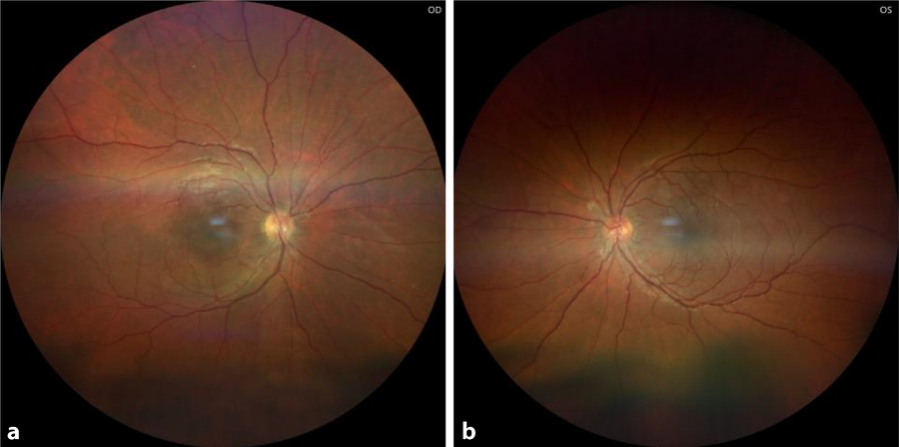

Im Rahmen der Pars-plana-Vitrektomie rechts konnten die Blutungen unter der ILM entfernt werden. Die subretinale sub- bzw. parafoveoläre Blutung war bereits depigmentiert und hat sich mittels präoperativer Konditionierung mit Alteplase als intravitreale, operative Medikamenteninjektion (IVOM) nicht lysieren lassen. Um dennoch eine Mobilisation der subretinalen Blutungsanteile zu erlangen, wurde die Netzhaut mittels 41-G-Katheter punktiert und eine kontinuierliche Makulaabhebung mit BSS („balanced salt solution“) induziert. Am Partnerauge (links) wurde bei singulärer Sub-ILM-Blutung auf eine präoperative Lyse mittels Alteplase-IVOM verzichtet. Intraoperativ gelang, nach dem Abpräparieren der ILM die Blutung mit der Backflush-Kanüle abzuspülen. Der Visus am Entlassungstag betrug rechts sc 0,2 (Glbn) und links sc 0,6 (Glbn), jedoch ohne Gesichtsfeldskotome. Bei der letztmaligen Nachkontrolle war der Visus identisch zum Entlasstag. Im Funduseinblick waren rechts bei wenigen zentralen Aufhellungen keine Blutungen nachweisbar; links zeigten sich regelrechte anatomische Verhältnisse (Abb. 3). Im OCT war rechts das subretinale Blut vollständig resorbiert, die korrespondierende Photorezeptorschicht befand sich im Stadium der Re-Organisation. Links zeigte das OCT parafoveal nasal eine punktuelle Degeneration der Photorezeptorschicht.

## Diskussion

In unserem Fallbericht sollen die Überdruckbeatmung in Bauchlage und COVID-19-assoziierte Gefäßveränderungen als Einflussfaktoren zur Entstehung einer Valsalva-Retinopathie bedacht werden.

### Potenzielle, bereits bekannte retinale Pathomechanismen durch SARS-CoV-2

**Figure 4:**
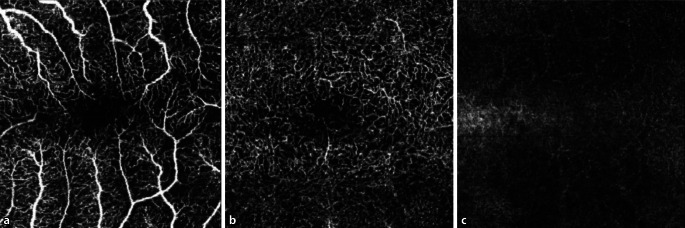

Beschreibungen zu Veränderungen der hinteren Augenabschnitte/der Retina im Rahmen von COVID-19 sind bislang rar [1]. COVID-19 kann mit einer systemischen Hyperkoagulabilität und sowohl Mikroangiopathie als auch mit lokaler Bildung von Thromben vergesellschaftet sein [2]. Auswirkungen einer SARS-CoV-Infektion auf die Retina sind daher eher sekundären Effekten der Multisystemerkrankung COVID-19 über mikrovaskuläre und thrombembolische Ereignisse zuzuschreiben [2]. Bislang konnte gezeigt werden, dass die Fovea-zentrierte oberflächliche und tiefe Gefäßdichte in der OCT-Angiographie bei mittleren und schweren COVID-Patienten signifikant reduziert ist [3, 4]. Die bei unserem Patienten präoperativ durchgeführte OCT-Angiographie zeigte links eine foveale avaskuläre Zone von 0,25 mm^2^, welche irregulär konfiguriert ist (Abb. 4 und 5). Die OCT-Angiographie rechts konnte bei Artefakten durch die subretinale Blutung nicht ausgewertet werden.

**Figure 5:**
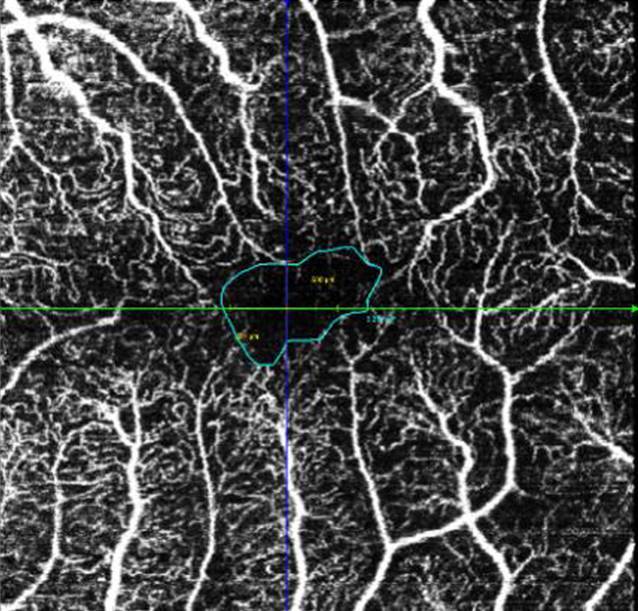

### Retinale Blutungen durch intravasale Druckerhöhung

Durch Duane wurde das Krankheitsbild der hämorrhagischen Valsalva-Retinopathie 1972 erstmals benannt [5]. Zuvor hat Byrnes (United States Air Force) 1959 durch hydrostatische Druckgradienten induzierte retinale Veränderungen (Blutungen) nach einer Schleudersitzejektion bei Überschallgeschwindigkeit aus einem Militärjet beschrieben. Der hierbei plötzlich anliegende, enorme Staudruck am Körper hatte eine extreme Valsalva-Situation über Kompression von Thorax und Abdomen zur Folge. Dieser Fallbericht beschreibt eindrücklich die einwirkende Energie auf die Körperoberfläche und deren Fortleitung über intravaskuläre Druckgradienten [6].

Insbesondere das Gehirn und das Auge als in knöcherne, nicht nachgiebige Körperhöhlen eingebettete Organe/neuronale Netzwerke scheinen besonders anfällig für vaskulär fortgeleitete, externe Energieeinwirkung zu sein [6]. Der normwertige intrakranielle Druck (im Liegen 8–10 mm Hg [7]) ist niedriger als der normwertige intraokulare Druck. Folglich kann bei Vorliegen einer Valsalva-Retinopathie die ursächliche venöse Druckspitze in zerebralen Gefäßen höher ausfallen, da der protektive Gegendruck geringer ist, als dies intraokulär der Fall ist. Daher könnte eine Bildgebung des Neurokraniums zum Ausschluss von relevanten, mikrovaskulären Veränderungen sinnvoll erscheinen.

Inwieweit und zu welchen Teilen die schwere COVID-Erkrankung, die Überdruckbeatmung oder die prolongierte Bauchlage zu oben beschriebenen retinalen Blutungen beigetragen haben, bleibt letztendlich offen.

## Fazit für die Praxis

- Die COVID-19-Pandemie führte zu einer großen Anzahl an in Bauchlage überdruckbeatmeten Patienten aller Altersgruppen. Bauchlage, Überdruckbeatmung, Pneumonie sowie Übergewicht können mit erhöhtem intrathorakalem Druck bei verschlossener Glottis (Valsalva-Situation) assoziiert sein.
- Bei Mehrung von Risikofaktoren für die Entstehung einer Valsalva-Retinopathie sollte eine Fundoskopie bzw. OCT-Diagnostik veranlasst werden.
- Eine kraniale Bildgebung kann bei Vorliegen einer Valsalva-Retinopathie zur Frühdetektion von zerebralen mikroangiopathischen Gefäßveränderungen wegweisend sein.
